# Bacteriophages as Vehicles for Antibiotic Resistance Genes in the Onyar River, Spain

**DOI:** 10.1007/s00248-025-02541-0

**Published:** 2025-05-08

**Authors:** Edgar González-Villalobos, Ana Carolina Maganha de Almeida Kumlien, Alexandre Sànchez-Melsió, José Luis Balcázar

**Affiliations:** 1 Catalan Institute for Water Research (ICRA-CERCA), 17003 Girona, Spain; 2 Research Division, Department of Public Health, Faculty of Medicine UNAM, 04510 Mexico City, Mexico

**Keywords:** Antibiotic resistance, ARGs, MGEs, Phages, Urban river, Sediments

## Abstract

This study aimed to investigate the presence and abundance of antibiotic resistance genes (ARGs) in bacterial and phage DNA fractions from sediment samples collected from the Onyar River, both before and after its passage through the urban area of Girona (northeast Spain). Genes conferring resistance to β-lactams, fluoroquinolones, macrolides, sulfonamides, and tetracyclines were quantified using quantitative PCR. Our findings showed that ARGs are present in both bacterial and phage DNA fractions, with a higher abundance in the bacterial fraction. Notably, our analysis revealed an increased abundance of the sulfonamide resistance gene *sulI* in the phage DNA fraction when comparing samples collected before and after the river’s passage through the city. Although similar trends were observed for other ARGs (e.g., *qnrS* and *sulII*), these differences were not statistically significant (*p* > 0.05). These findings emphasize the importance of phages as potential reservoirs or vehicles for ARGs in environmental settings. Further research is needed to elucidate the factors that influence gene transfer dynamics and the persistence of ARGs within phages.

**Supplementary Information:**

The online version contains supplementary material available at 10.1007/s00248-025-02541-0.

The growing prevalence of antibiotic resistance represents a major challenge to antibiotic therapy, as it compromises the efficacy of prescribed treatments and poses a critical threat to public health worldwide [1]. Recent estimates suggest that approximately 1.3 million deaths occur annually as a direct consequence of antibiotic resistance [2], and current trends indicate that this number could rise to 1.91 million attributable deaths and 8.22 million associated deaths by 2050 [3]. These data highlight the urgent need for coordinated, comprehensive, and sustained efforts to contain the spread of antibiotic resistance and mitigate its impact on global health.

Addressing this challenge requires the implementation of strategies guided by the One Health approach, which recognizes the interconnectedness of human, animal, and environmental health. Within this framework, aquatic ecosystems are increasingly recognized as critical environments that contribute to the emergence, persistence, and dissemination of antibiotic resistance across both environmental and clinical settings [4].

Although antibiotic resistance is a naturally occurring phenomenon in bacterial populations, the widespread and continuous discharge of antibiotic residues into aquatic environments, including untreated and treated domestic wastewater, agricultural runoff, and industrial discharges, has significantly exacerbated the problem. These residues exert selective pressure on microbial communities, promoting the survival, proliferation, and dissemination of antibiotic-resistant bacteria, including clinically relevant pathogens.

Urban rivers, in particular, are continuously exposed to a wide range of chemical and biological contaminants, including antibiotic residues, heavy metals, and antibiotic-resistant bacteria from anthropogenic sources [5]. These conditions create environments that are highly favorable for the exchange of genetic material among bacterial communities, including antibiotic resistance genes (ARGs).

A key mechanism driving the spread of these ARGs is horizontal gene transfer (HGT), which enables bacteria to acquire foreign genetic material both intra- and interspecifically [6]. This process is mediated by mobile genetic elements (MGEs), such as insertion sequences, transposons, plasmids, genomic islands, and bacteriophages (phages). Among the different mechanisms of HGT, conjugation is widely recognized as the primary pathway for the dissemination of ARGs, due to its high frequency and its capacity to mediate plasmid transfer across a broad spectrum of bacterial species [7, 8]. Despite their ubiquity, the role of phages in the environmental dissemination of ARGs remains poorly understood. Phages, viruses that infect and replicate within bacterial cells, are widespread and highly abundant in diverse ecosystems [9]. Through their interactions with bacterial hosts, phages play a crucial role in bacterial evolution and ecology [10–12], positioning them as potential vehicles for the dissemination of ARGs.

Given the limited research on the role of phages in mobilizing ARGs in the environment, this study aimed to detect ARGs in the Onyar River, an urban river that flows from south to north through the city of Girona, in northeast Spain. The selection of target genes was based on their clinical and environmental relevance, as well as their potential use as indicators of antibiotic pollution in the environment [13, 14]. The selected genes included those conferring resistance to key antibiotic classes: β-lactams (*bla*_CTX-M_ and *bla*_KPC_), fluoroquinolones (*qnrS*), macrolides (*ermB*), sulfonamides (*sulI* and *sulII*), and tetracyclines (*tetW*). These ARGs were then quantified by quantitative PCR (qPCR) in both the phage-derived and bacterial DNA fractions from sediment samples for comparative analysis.

Three sediment samples (50 g each) were collected from the Onyar River, both before and after its flow through the city of Girona. To ensure representative sampling, three locations were selected along each transect: one on the right bank, one on the left bank, and one in the center of the river. The samples were immediately transported to the laboratory, resuspended in Ringer’s solution, and vortexed for 15 min. The supernatants were subsequently filtered through 0.22-μm pore-size membranes, allowing phage particles to pass into the filtrate while retaining bacterial cells on the filter surface. The retained bacterial cells were resuspended in Tris–EDTA buffer and digested with lysozyme (40 mg/ml) and proteinase K (20 mg/ml) prior to DNA extraction, which was carried out using a standard phenol–chloroform method [15]. The filtrates containing phage particles were precipitated using polyethylene glycol (PEG 6000), centrifuged at 14,000 g for 10 min, and the resulting pellets were treated twice with DNase (100 U/ml) to remove free DNA outside the phage particles. Subsequently, phage DNA extraction and purification were performed as previously described [16]. DNA concentration was measured using a Qubit 2.0 fluorometer (Life Technologies; Carlsbad, CA, USA). Moreover, the phage DNA fraction was screened for the presence of 16S rRNA genes by qPCR [17], as the abundance of ARGs in phages can be overestimated due to bacterial DNA contamination [18, 19].

The copy number of the target ARGs was determined by qPCR. All reactions were performed using a CFX96 Touch Real-Time PCR Detection System (Bio-Rad Laboratories; Hercules, CA, USA) under previously described conditions [20]. To ensure amplification specificity, a dissociation curve was generated for each reaction, ranging from 60 to 95 °C. The efficiency and sensitivity of the qPCR assays were assessed by constructing standard curves from ten-fold serial dilutions of synthetic gene fragments at known concentrations (Integrated DNA Technologies; Coralville, IA, USA). Nuclease-free water and qPCR master mix were included as negative controls, while synthetic ARG sequences were used as positive controls to validate the reactions.

The qPCR data were tested for normality using the Shapiro–Wilk test and for homoscedasticity using Levene’s test (see Supplementary Figure S1). When the assumptions of normality and homogeneity of variance were met, mean values from three replicates per gene were compared using one-way analysis of variance (ANOVA) followed by Tukey’s HSD test. When these assumptions were not met, the non-parametric Kruskal–Wallis test followed by Dunn’s post hoc test was applied. These statistical analyses were used to assess significant differences (*p* < 0.05) among DNA fractions from sediment samples. All analyses were performed using the *ggpubr* and *rstatix* packages in R (v4.4.3; R Core Team, 2025).

Although phages can transfer genetic material between bacterial hosts via transduction, the extent to which this occurs in environmental settings remains controversial, primarily due to concerns about the potential overestimation of phage-encoded ARGs caused by bacterial DNA contamination [19, 21, 22]. To address this, all phage DNA fractions were initially screened for the presence of 16S rRNA genes to assess possible bacterial contamination. The results showed that none of the phage DNA fractions contained detectable levels of bacterial DNA contamination, confirming their suitability for subsequent analysis of ARG abundance. Both DNA fractions were then analyzed by qPCR, which demonstrated higher copy numbers of ARGs in the bacterial DNA fraction compared to the phage DNA fraction, with statistically significant differences observed for some ARGs.

All ARGs were detected in the analyzed samples, except for the *bla*_KPC_ gene, which was found exclusively in the bacterial DNA fraction from sediment samples collected after the river’s passage through the urban area (Fig. 1). Moreover, with the exception of the *bla*_CTX-M_ gene, higher copy numbers of ARGs were observed in the bacterial DNA fraction from samples collected after the river had entered the city, compared to those collected before its passage. These findings suggest that the studied river is influenced by anthropogenic pollution. Although the river does not receive direct urban wastewater discharges during its passage through the city, previous studies have indicated that surface runoff may contribute to the presence of ARGs, thereby facilitating the dissemination of antibiotic resistance [23, 24].

**Fig. 1:**
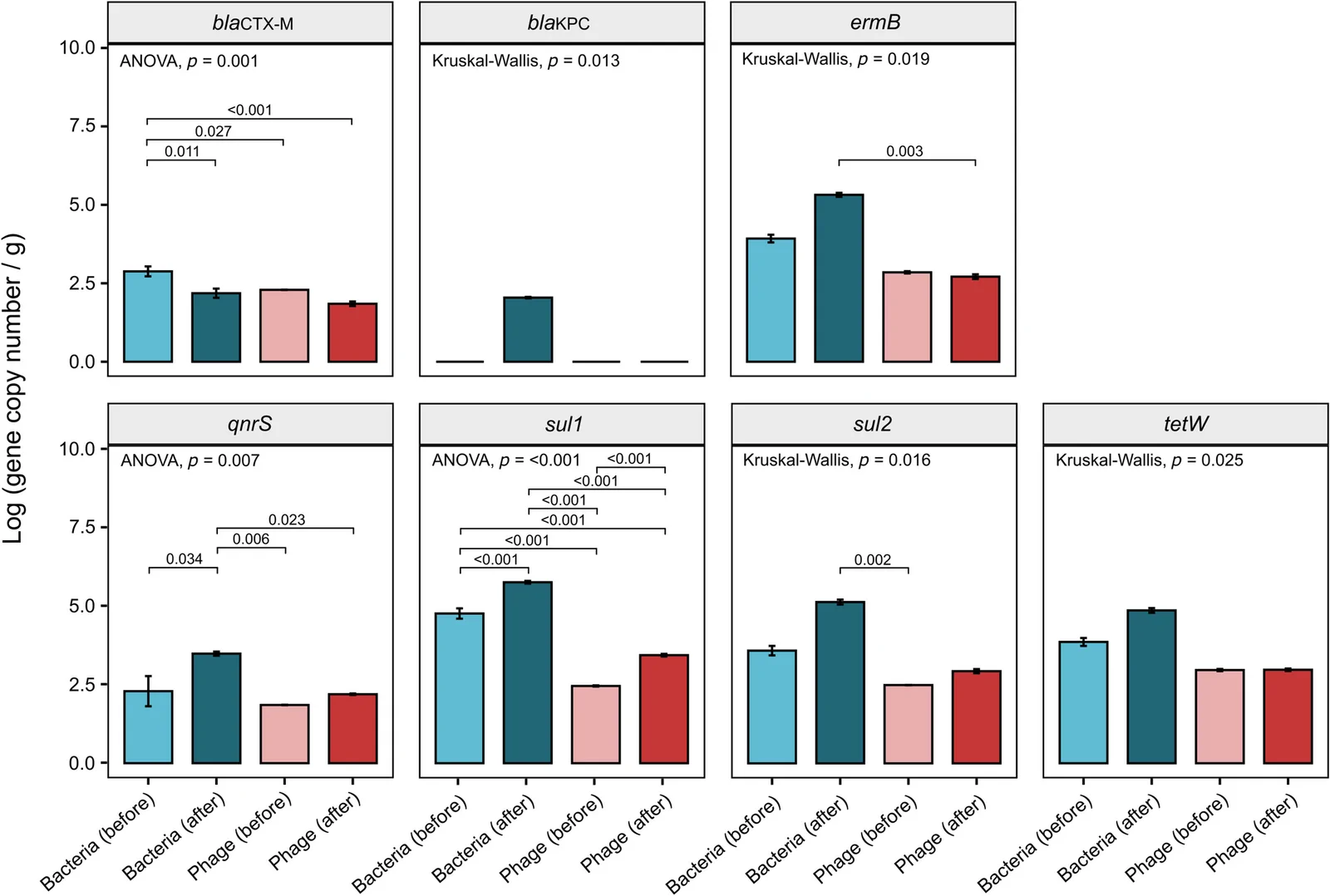
Copy numbers of ARGs in bacterial and phage DNA fractions from sediment samples (per gram) collected from the Onyar River, before and after its passage through the urban area of Girona. Data were log_10_-transformed prior to statistical analysis using ANOVA or the Kruskal–Wallis test, as appropriate. Bar colors represent DNA fractions (bacterial or phage). Values are presented as means (*n* = 3) ± standard deviation

Interestingly, our analyses also revealed a tenfold increase in the abundance of sulfonamide resistance genes (*sulI* and *sulII*) in the phage DNA fraction from sediment samples collected after the river’s passage through the city. However, statistically significant differences (< 0.001) were observed only for the *sulI* gene. These findings are consistent with previous studies, which suggest that sulfonamide resistance genes are among the most commonly used tracers for assessing ARGs due to their strong correlation with anthropogenic inputs in environmental settings [14, 25]. In contrast, the abundance of the remaining ARGs did not exhibit any significant differences in the phage DNA fraction between samples collected before and after the river’s passage through the city (Fig. 1). While there were no significant differences in the abundance of genes conferring resistance to β-lactam antibiotics (*bla*_CTX-M_), fluoroquinolones (*qnrS*), macrolides (*ermB*), and tetracyclines (*tetW*) between samples collected before and after of the urban area, their presence in phages raises environmental and public health concerns. In fact, a recent study reported that genes conferring multidrug and β-lactam resistance are present in temperate phages from a lake entirely replenished with reclaimed water. The study also found positive correlations between phages harboring ARGs and host pathogens, suggesting the potential emergence of antibiotic-resistant pathogens [26]. Additionally, a metagenomic analysis of influent and effluent samples from a wastewater treatment plant in China revealed that although the treatment process reduced the overall abundance of ARGs, some ARGs remained detectable. Notably, certain ARGs were associated with phages, and a higher presence of MGEs was observed in the effluent, indicating an increased potential for the persistence and spread of ARGs within wastewater treatment plants [27].

A recent study also demonstrated the presence of ARGs (*bla*_TEM_, *bla*_SHV_, *bla*_CTX-M_, *bla*_CMY_, *mecA*, *vanA*, and *mcr*−1) in the phage fraction of urban and hospital wastewater samples, with *bla* genes detected at the highest frequency, while *mecA* and *mcr*−1 were the least frequently observed [28]. These findings highlight the role of phages as reservoirs of ARGs, with significant public health implications due to their environmental stability and persistence.

Taken together, our results demonstrate that phages harbor ARGs, although to a lesser extent than bacteria. Thus, they can act as potential reservoirs or vehicles for ARGs in environmental settings, contributing to their persistence and dissemination. Given the global distribution of phages and their greater persistence compared to bacteria during disinfection processes, their role in the spread of ARGs should not be underestimated. These considerations are essential for the development and implementation of effective surveillance and mitigation strategies to address the growing global crisis of antibiotic resistance.

## Supplementary Information

Below is the link to the electronic supplementary material.

ESM1 Assessment of statistical assumptions underlying parametric and non-parametric analyses. Data were tested for normality using the Shapiro–Wilk test and for homoscedasticity using Levene’s test prior to statistical analysis. These tests were performed to determine the suitability of ANOVA or the Kruskal–Wallis test for comparing ARG copy numbers between bacterial and phage DNA fractions. (PNG 410 KB) 

High Resolution Image (671 KB)

## Data Availability

The data that support the findings of this study are available from the corresponding author, upon reasonable request.
