# Supplementary figures and images for: Bacteriophages as Vehicles for Antibiotic Resistance Genes in the Onyar River, Spain
Source: Microb Ecol. 2025 May 8;88(1):41. doi: 10.1007/s00248-025-02541-0 (PMC12089252; doi:10.1007/s00248-025-02541-0)

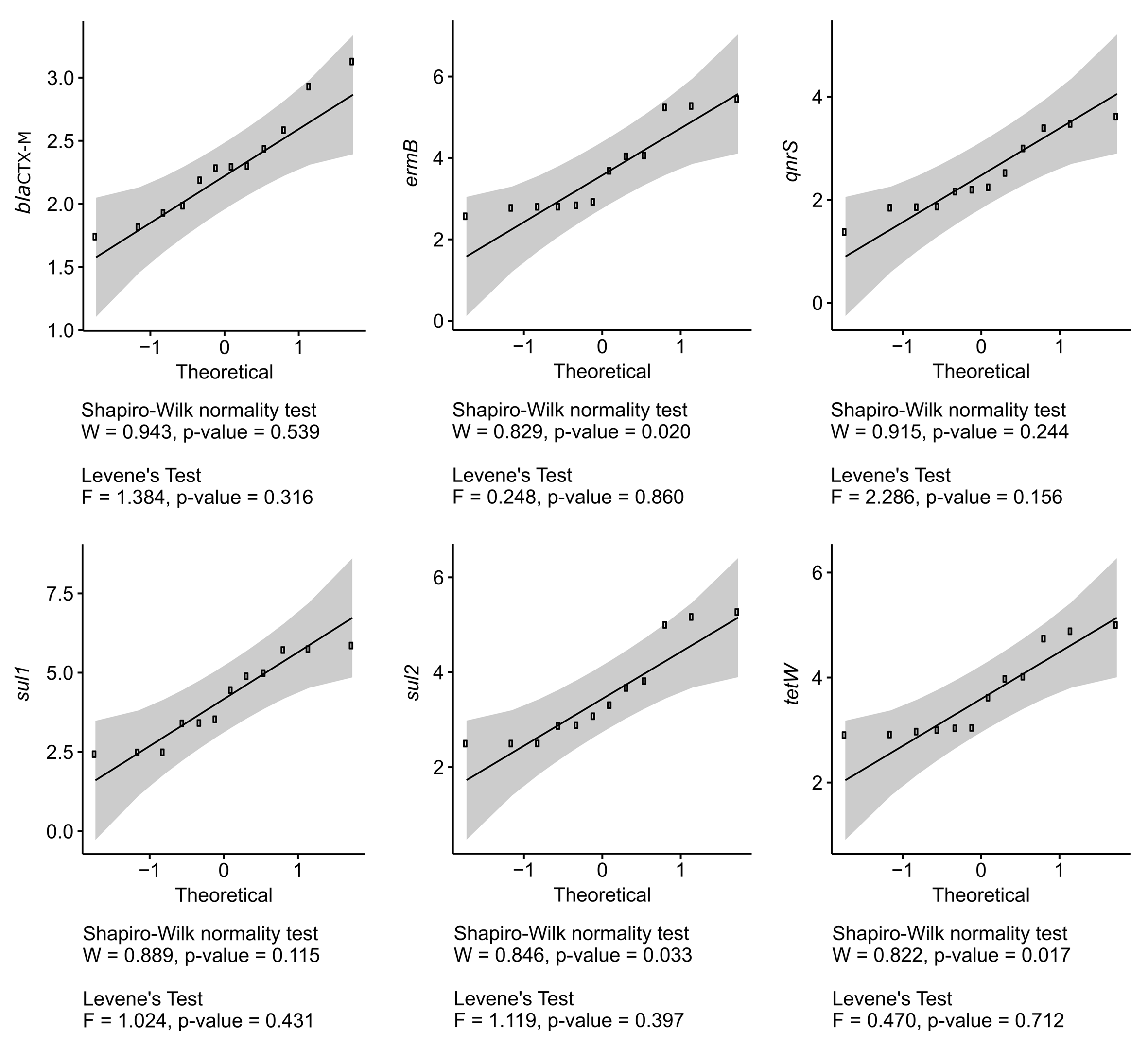

Supplement: Supplementary file 1 — Assessment of statistical assumptions underlying parametric and non-parametric analyses. Data were tested for normality using the Shapiro–Wilk test and for homoscedasticity using Levene’s test prior to statistical analysis. These tests were performed to determine the suitability of ANOVA or the Kruskal–Wallis test for comparing ARG copy numbers between bacterial and phage DNA fractions. (PNG 410 KB) [file 248_2025_2541_Fig2_ESM.png]

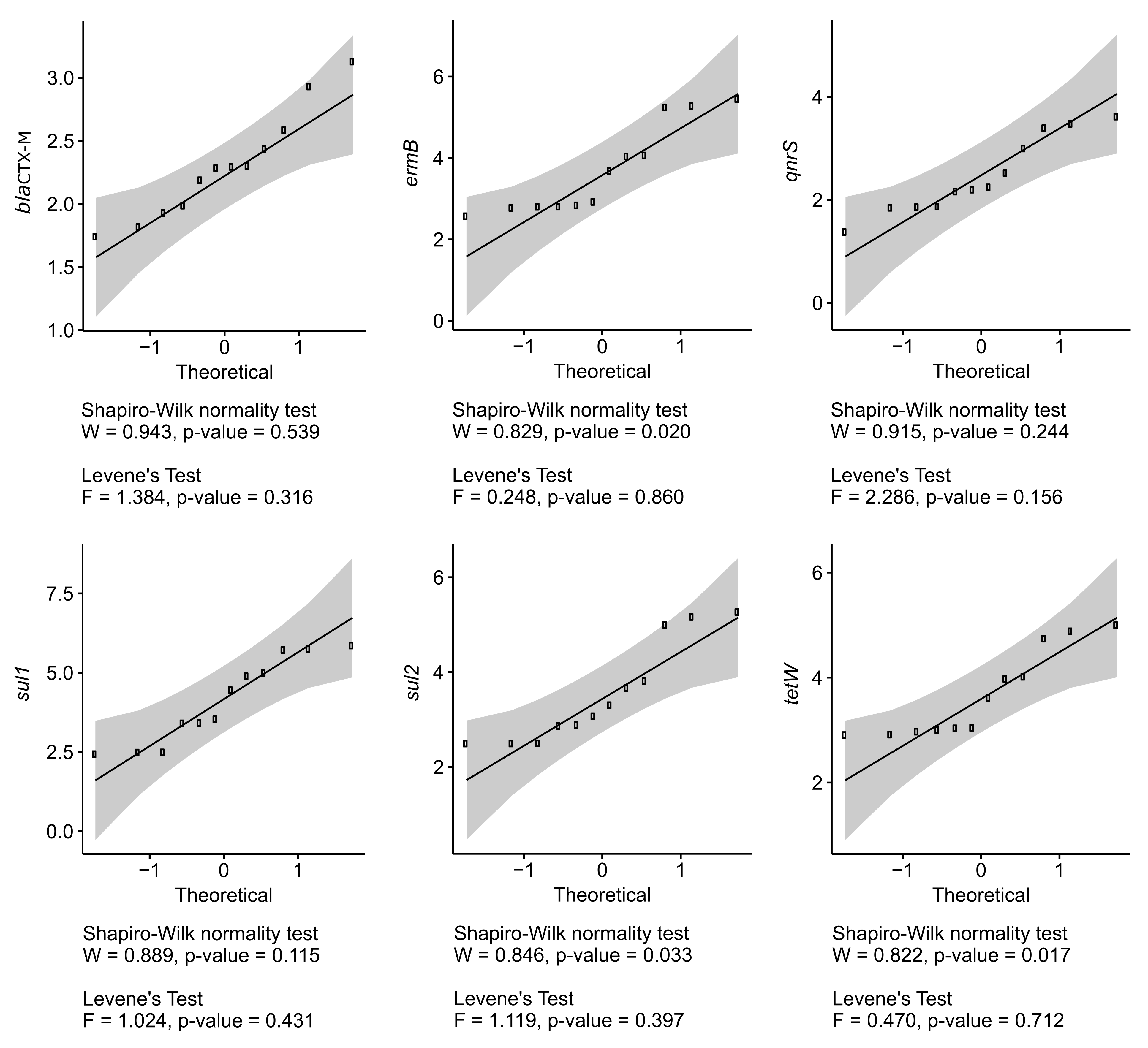

Supplement: Supplementary file 2 — High Resolution Image (671 KB) [file 248_2025_2541_MOESM1_ESM.tif]
